# Detecting subtle signs of depression with automated speech analysis in a non-clinical sample

**DOI:** 10.1186/s12888-022-04475-0

**Published:** 2022-12-27

**Authors:** Alexandra König, Johannes Tröger, Elisa Mallick, Mario Mina, Nicklas Linz, Carole Wagnon, Julia Karbach, Caroline Kuhn, Jessica Peter

**Affiliations:** 1 Institut National de Recherche en Informatique Et en Automatique (INRIA), Sophia Antipolis, Stars Team, Valbonne, France; 2 Ki Elements, Saarbrücken, Germany; 3 University Hospital of Old Age Psychiatry and Psychotherapy, University of Bern, Bolligenstrasse 111, CH-3000 Bern 60, Switzerland; 4 Department of Psychology, University of Koblenz-Landau, Koblenz, Germany; 5 Department of Psychology, Clinical Neuropsychology, University of Saarland, Saarbrücken, Germany

**Keywords:** Depressive symptoms, Automated speech analysis, Acoustic features, Textual features, Machine learning

## Abstract

**Background:**

Automated speech analysis has gained increasing attention to help diagnosing depression. Most previous studies, however, focused on comparing speech in patients with major depressive disorder to that in healthy volunteers. An alternative may be to associate speech with depressive symptoms in a non-clinical sample as this may help to find early and sensitive markers in those at risk of depression.

**Methods:**

We included *n =* 118 healthy young adults (mean age: 23.5 ± 3.7 years; 77% women) and asked them to talk about a positive and a negative event in their life. Then, we assessed the level of depressive symptoms with a self-report questionnaire, with scores ranging from 0–60. We transcribed speech data and extracted acoustic as well as linguistic features. Then, we tested whether individuals below or above the cut-off of clinically relevant depressive symptoms differed in speech features. Next, we predicted whether someone would be below or above that cut-off as well as the individual scores on the depression questionnaire. Since depression is associated with cognitive slowing or attentional deficits, we finally correlated depression scores with performance in the Trail Making Test.

**Results:**

In our sample, *n =* 93 individuals scored below and *n =* 25 scored above cut-off for clinically relevant depressive symptoms. Most speech features did not differ significantly between both groups, but individuals above cut-off spoke more than those below that cut-off in the positive and the negative story. In addition, higher depression scores in that group were associated with slower completion time of the Trail Making Test. We were able to predict with 93% accuracy who would be below or above cut-off. In addition, we were able to predict the individual depression scores with low mean absolute error (3.90), with best performance achieved by a support vector machine.

**Conclusions:**

Our results indicate that even in a sample without a clinical diagnosis of depression, changes in speech relate to higher depression scores. This should be investigated in more detail in the future. In a longitudinal study, it may be tested whether speech features found in our study represent early and sensitive markers for subsequent depression in individuals at risk.

**Supplementary Information:**

The online version contains supplementary material available at 10.1186/s12888-022-04475-0.

## Background

Major depression is a rising mental health concern, affecting more than 264 million people worldwide [1]. Detecting depressive symptoms as early as possible becomes increasingly important in younger adults as mental health concerns are rising in this population, particularly since the COVID-19 pandemic [2]. The analysis of speech offers a promising avenue to objectively identify signs of depression as the cognitive and behavioural changes associated with depression influence the production as well as the quality of speech [3, 4]. Patients with depression typically present with decreased or less fluent speech, with diminished prosody, or with monotonous speech [3, 5]. It is, therefore, not surprising that clinicians either consciously or unconsciously watch for signs of changed speech during the diagnostic process of this disorder. The subjective evaluation of a change in speech, however, is open to bias and requires a large degree of clinical training to produce reliable results. In addition, subtle changes may remain undetected. Thus, clinicians would greatly benefit from objective measures to identify impairment in speech control or the acoustic quality of the speech produced. During recent years, automated analysis of speech has gained increasing attention and paralinguistic features have been investigated in particular [6, 7]. These features convey or modify meaning beyond the words and grammar used and include pitch, prosody, volume, or intonation (among others) and can be considered a key behavioural marker of depression [3]. Early studies investigating paralinguistic features found that patients with depression consistently demonstrated prosodic speech abnormalities such as reduced pitch, reduced pitch range, slower speaking rate and articulation errors [8–10]. Others identified decreased intonation, slow articulation and vocal monotony in patients with depression [3]. A recent review on studies using automated speech analysis added that changes in fundamental frequency, jitter, and shimmer have been repeatedly reported to be associated with depression [7]. Automated speech analysis, therefore, has made it possible to study and compare speech features on even finer scales. Most of the studies so far, however, focused on discovering speech features associated with depressive symptoms by comparing healthy volunteers to patients with major depression. In order to find early and sensitive markers for possible preventive strategies, however, it may be useful to also consider a ‘healthier’ population. In addition, a timely detection of psychological distress is particularly crucial in younger adults since a depressive episode will follow if no support is provided [11]. The objective of this study was, therefore, to test whether there would be an association between subtle signs of depression and speech features in a non-clinical population (i.e., in healthy young adults). Since the major cognitive concerns in depression are deficits in controlling attention due to rumination, cognitive slowing, and problems with executive control [12, 13], we also tested whether subtle signs of depression would relate to attentional deficits, cognitive slowing, or executive function deficits.

## Materials and methods

### Participants

We initially recruited *n =* 164 healthy young University students (20–30 years of age, 79% women) from the University of Saarbrücken or the University of Koblenz-Landau (both in Germany). The study procedures were similar at both universities. All participants gave written informed consent prior to study participation. All experiments were done in accordance with the ethical standards of the Universities of Koblenz-Landau and Saarbrücken.

### Study procedure

The participants completed the Trail Making test (see below) on a tablet in a lab environment at the University of Saarbrücken or the University of Koblenz-Landau. Afterwards, the participants were asked to talk about a negative and a positive event in their life. We standardized the recording procedure by using an iOS-based iPad Pro A1584, with a 12.9’ display and a resolution of 2732 × 2048 pixels. The procedure always took place in the same room with identical settings: Noise-cancelled surrounding, standard-lightings, iPad lying on the table (in near visual acuity distance; i.e., 35–40 cm) and was operated by the (diagonally opposite sitting) examiner. Finally, we examined symptoms of depression with a self-report questionnaire (see below). All participants received course credit for their participation.

### Trail making test (A and B)

With the Trail Making Test [14] A and B, we assessed speed of information processing, focused or divided attention, and switching abilities. For the Trail Making Test A, the participants were asked to connect randomly positioned numbered circles (1–25) in ascending order as quickly as possible. For the Trail Making Test B the participants needed to connect circles that included numbers (1–13) or letters (A-L) in numeric and alphabetic order as quickly as possible, alternating between numbers and letters. We used time to completion for statistical analysis.

### Depression scale

For the assessment of depressive symptoms, we used a reliable and valid questionnaire (‘Allgemeine Depressionsskala’), which assesses disturbances caused by depressive symptoms during the last week [15]. For the total score (range between 0–60), the frequency of motivational, emotional, somatic, or cognitive symptoms (20 items in total) had to be rated on a scale ranging from 0 (never or seldom) to 4 (mostly or all the time). Based on the total score, participants were classified as either above (score ≥ 22) or below (score < 22) the cut-off for clinically relevant depressive symptoms [15].

### Speech task (positive and negative story)

Free and natural speech tasks are capable of eliciting emotional reactions (or a lack thereof) by asking to describe events that triggered recent affective arousal. To this end, and based on our previous research [16, 17], the participants were asked to talk about a positive and a negative event in their life. Instructions for the speech tasks (‘Can you tell me in one minute about a positive/negative event in your life?’) were pre-recorded by psychologists and played to the participants from a tablet ensuring standardized instructions. The answers were recorded with the tablet’s microphone.

### Processing of speech data

Recordings of the positive and negative story were transcribed by two Psychology students (M. Sc.) according to the CHAT protocol [18]. Audio features were automatically extracted from the audio signal. Textual features were automatically extracted from the manual transcripts. We used the proprietary speech processing engine SIGMA [19] to extract textual and audio features. Features were extracted separately for positive and negative stories. These include acoustic features with segmental features (e.g., the number or length of words or pauses or the number or length of speaking segments), supra-segmental or prosodic features based on the processing of the frequency spectrum (e.g., pitch, intensity), and textual features. Acoustic features were calculated only for the parts when the participants told the positive/negative story. Feature extraction was similar as in our previous studies [16, 17]. Different software packages were utilized to calculate the features. The computation of fundamental frequency related features relied on the parselmouth package [20] (which is a python wrapper for praat; [21]). The pitches were computed with the standard value (i.e., zero) for time step, 50 Hz for pitch floor, and 250 Hz for pitch ceiling. The WebRTC Voice Activity Detector (py-webrtcvad; https://github.com/wiseman/py-webrtcvad) package was used to estimate pause and speech ratio. A frame size of 10 ms was used to partition the audio data before marking them as pause or speech. A threshold of 1 s was used to determine whether consecutive frames form meaningful speech. Most of the linguistic features relied on the stanza package [22] with the default tokenization. The computation of the rates of parts of speech (POS) is straightforward with stanza's provided POS-tagging. Graph related features were calculated with the NetworkX package [23]. Semantic clusters were computed using transcript's word embedding (provided by the flair package [24]) by applying sklearn's affinity propagation. More precisely, word vectors of the tokens in the transcripts (without any processing like stemming) were computed using word embedding provided by the flair package. Then affinity propagation clustering on word vectors was computed with default Euclidean affinity and default damping of 0.5. The coherence metric was estimated following the work by Iter and colleagues [25]: the cosine similarity between consecutive sentences was computed and then the mean of the similarity value was taken. Sentiment related features were computed with the sentiment intensity analyser of the nltk package [26]. A list of all extracted features with an explanation of their meaning can be found in supplementary Tables S1 and S2. Since speech features vary naturally between men and women, we normalized features by gender (i.e., they were scaled by their minimum and maximum absolute value, by gender).

### Statistical analysis

Our first aim was to test whether speech features differ between individuals below and above the cut-off of clinically relevant depressive symptoms. Since previous studies found that patients with depression used more words than healthy volunteers in written self-reports [27], we first compared the number of words in the positive and the negative story between groups, using multivariate analysis of variance with group as between-subject factor and the number of words as dependent variable. Next, we tested whether there were any differences in speech features between individuals below and above the cut-off using multivariate analysis of variance, with group (below or above cut-off) as between-subject factor and speech features as dependent variables, when controlling for the number of words in each story. Next, we performed logistic regression to test whether speech features can predict whether or not a person would score below or above the cut-off. We used binary logistic regression with backward selection using Wald statistics (probability of F for entry: p ≤ 0.05, probability of F for removal: p ≥ 0.10). Then, we predicted the individual depression scores using speech features and machine learning based regression models (i.e., support vector machine, extra trees, or random forest). These were independently trained on 322 extracted features (i.e., 161 features for each story, positive and negative). We used leave-one-out cross validation and grid search for hyper parameter tuning. We trained each of the three models (support vector machine, extra tree, random forest) 118 times and selected the best model based on the lowest Mean Absolute Error. In addition, we tested the significance of each model by comparing its’ performance to an extra trees model trained while permuting the true depression scores 1000 times. Next, we tested whether we would find significant differences in Trail Making Test performance between individuals below and above cut-off using multivariate ANOVA, with group as between-subject factor and performance as dependent variable. Finally, we correlated, in each group (i.e., below or above cut-off), the scores achieved in the depression score with time to complete the Trail Making Test (A and B), using Pearson correlation or Spearman correlation (to account for potential outliers).

We used SPSS (version 26.0; IBM Inc.; USA) and R (version 1.4.1106) for statistical analyses and GraphPad Prism (version 9.0.0; USA) for visualization of the results. Statistical significance levels were set to *p* < 0.05 (two-tailed). We adjusted for multiple comparisons using the Bonferroni-Holm method.

## Results

We had to exclude 44 participants due to missing data (data was missing at random). We additionally excluded two participants whose answers on the depression questionnaire indicated a response bias. One participant was classified as an age outlier (> 3 SD above mean) and was therefore also excluded. Thus, a total of *n =* 118 was included in all statistical analyses, of whom *n =* 93 were below the cut-off of relevant depressive symptoms and *n =* 25 were above that cut-off. We did not observe significant differences regarding age, sex-ratio, or education between these two groups (Table 1). In neither of the two groups, we found a significant difference between males and females in the depression scores.

**Table 1:** Demographics of the sample when divided into participants above cut-off for relevant depressive symptoms and below that cut-off. We used *Χ*^2^ (sex) or ANOVA (all other variables) to test for significant differences between both groups

	Non clinical sample (*n =* 118)		
	Below cut-off (*n =* 93)	Above cut-off (*n =* 25)	*p*-value
Age (years)	23.5 ± 3.8	23.4 ± 3.2	0.86
Sex (men/women)	24/69	3/22	0.19
Education (years)	15.3 ± 2.2	15.6 ± 2.4	0.56
Depression score	15.9 ± 2.9	26.5 ± 4.7	< 0.001

*ANOVA* Analysis of variance

**Fig. 1:**
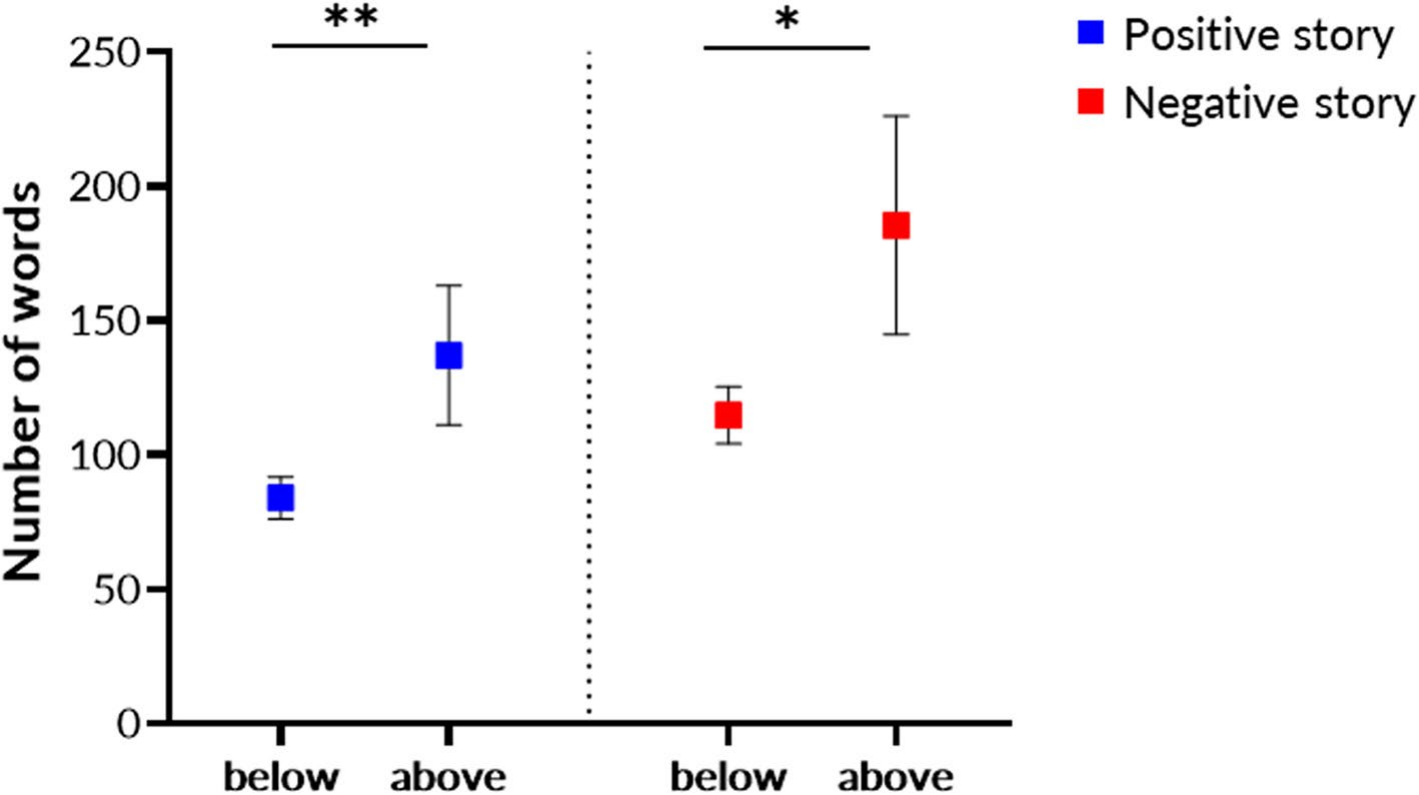
Number of words in a positive (blue) or negative (red) story in participants that were either below or above the cut-off of clinically relevant depressive symptoms

When comparing the number of words in the positive or negative story between both groups, we found that individuals in the above cut-off group spoke more than individuals below cut-off (*F*_(2, 115)_ = 3.71, *p* = 0.028) in both the positive (*F*_(1, 116)_ = 6.94, *p* = 0.01) and the negative story (*F*_(1, 116)_ = 5.83, *p* = 0.02) (Fig. 1). When comparing other speech features between groups, we did not find any significant difference for features obtained from the positive story *F*_(1, 115)_ = 1.13, *p* = 0.65) or the negative story *F*_(1, 115)_ = 57.29, *p* = 0.11), when controlling for the number of words in each story. When predicting whether a person would be below or above the cut-off of clinically relevant depressive symptoms using logistic regression (backward method), we found that the logistic regression model was statistically significant χ2 _(18)_ = 25.26, *p* < 0.0001. The model explained 78.4% of the variance (Nagelkerke R^2^) and correctly classified 93.2% of cases (Table 2).

**Table 2:** Classification table for the results of the binary logistic regression model. (i.e., prediction whether a person would be below or above cut-off for clinically meaningful depressive symptoms)

	Predicted	
Observed	Below cut-off	Above cut-off
Below cut-off	89	4
Above cut-off	4	20

The variables significantly contributing to the prediction model are presented in Table 3. When predicting scores in the depression questionnaire, best performance was achieved with a support vector machine (mean absolute error = 3.90) which was significantly better than the prediction of the random models (mean absolute error = 4.43, standard deviatio*n =* 0.17; *p* < 0.05).

**Table 3:** Variables that significantly contributed to explained variance in the binary logistic regression model (i.e., prediction whether a person would be below or above cut-off for clinically relevant depressive symptoms)

Predictor	Coefficient	Standard error	Wald	*p*-value
Speech ratio negative	426.79	169.30	6.36	0.012
Mean f0 positive	-0.55	0.22	6.26	0.012
Standard deviation f0 positive	-0.64	0.25	6.57	0.010
H1 a3 harmonic difference positive	-0.24	0.09	7.02	0.008
Average mfcc 2 negative	0.17	0.09	3.54	0.06
Average mfcc 3 negative	0.53	0.20	6.77	0.009
Average mfcc 5 negative	-0.98	0.34	8.27	0.004
Average mfcc 7 negative	-1.26	0.45	7.66	0.006
Average mfcc 7 positive	1.32	0.46	8.16	0.004
Mean pause duration positive	-18.34	7.19	6.50	0.011
Local jitter positive	27.19	11.53	5.56	0.018
Local absolute jitter positive	-6326.19	2537.08	6.22	0.013
Power spectrum ratio negative	89.27	32.68	7.46	0.006
Mean power positive	-5389.42	2067.18	6.79	0.009
Intensity standard deviation negative	1.04	0.40	6.63	0.010
Apq5 shimmer positive	-2.35	1.05	4.99	0.025
Local shimmer negative	2.57	0.97	6.96	0.008
Average dependency distance positive	6.60	2.53	6.79	0.009

*h1 a3 harmonic difference* Amplitude difference between first harmonic and third formant, *mfcc* Mel frequency cepstral coefficient, *apq5* Shimmer (5-point window)

**Fig. 2:**
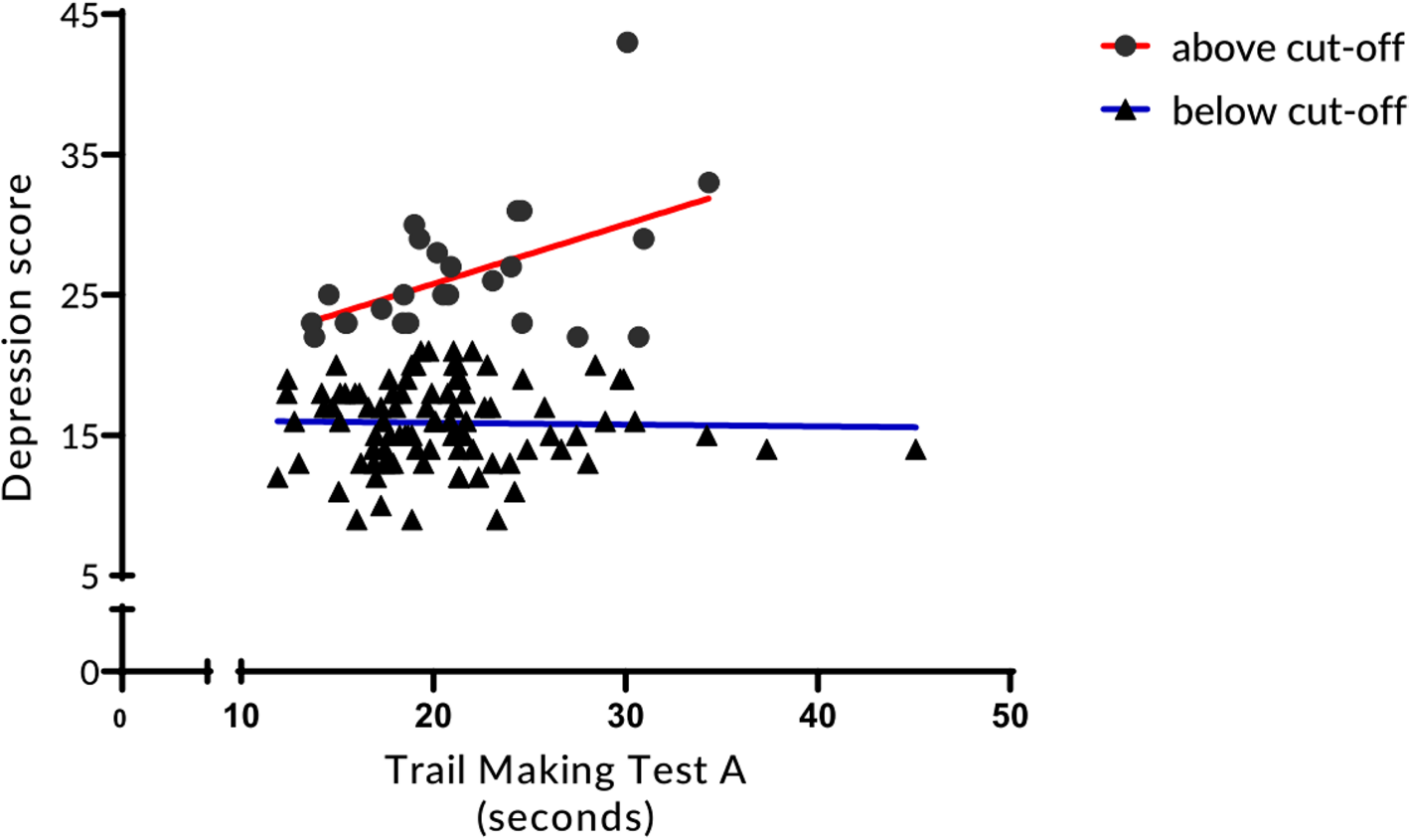
Correlation between the time it took to perform the Trail Making Test (part A) and scores in a depressive symptoms questionnaire in participants above or below cut-off of clinically relevant depressive symptoms

Finally, we tested whether there were any significant differences in Trail Making Test performance and whether performance correlated significantly with scores of depressive symptoms in each group. We found no significant difference between groups for Trail Making Test performance (*F*_(2, 115)_ = 0.78, *p* = 0.46). However, in the above cut-off group, those with higher depression scores were slower in performing the Trail Making Test A, as indicated by a significant positive correlation between performance and the depression score (*r*_(25)_ = 0.52, *p* = 0.008; Fig. 2). We neither found a significant correlation in the group below cut-off (*r*_(93)_ = -0.02, *p* = 0.87; Fig. 2) nor with performance in the Trail Making Test B (above cut-off: *r*_(25)_ = 0.28, *p* = 0.18; below cut-off: *r*_(93)_ = 0.18, *p* = 0.07). The results were similar when using non-parametric correlations (i.e., Spearman rank correlations).

## Discussion

In this study, young University students talked about a positive and a negative event in their life and we tested whether speech features in either story would be associated with the amount of depressive symptoms. We first classified participants as either above or below cut-off of clinically relevant depressive symptoms and tested whether individuals above or below cut-off would differ in the number of words used in either story. This was the case as participants above cut-off spoke significantly more than participants below cut-off in both the positive and the negative story (Fig. 1). This is contrary to previous findings in patients with manifest depression, which tended to speak less than healthy volunteers [5]. Our sample, however, was not considered pathological (i.e., clinically depressed) and thus, clinical signs of depression may have been too subtle to show speech patterns typically associated with manifest depression. This is supported by the mean score in the above cut-off group which was 26.5 (± 4.7). Since the cut-off for meaningful depressive symptoms is 22, the participants in the above cut-off group seem only mildly affected. An alternative explanation may be that along the dimensionality of depressive symptoms (i.e., from subtle signs to manifest depression), the number of words used to describe or tell something may change from very much to very little. This is supported by studies that included patients with mild symptoms of depression, who also used more words than healthy volunteers in written self-reports or in social media posts [27, 28]. It could be that the increase in the number of words in our participants, as well as in patients with mild depression, indicate rumination or ‘mind-wandering’, reflected by an increase in verbalized thought production [29]. Rumination is a predisposing factor contributing to an increased risk for developing major depression [30]. Therefore, it could be that our participants above the cut-off are at risk for major depression in the future. Interestingly, participants above cut-off appeared to produce more words in the negative story than in the positive story (Fig. 1, non-significant finding), which is in line with previous studies in patients with manifest depression [31]. Our findings therefore indicate that even in a non-clinical sample, those with symptoms of depression produce more words when a negative cue is given. Another possible explanation may be that the increase in word count indicates loosening of associations. By increasing the number of words, the individuals may compensate for distraction, cognitive slowing or even for hesitancy.

We did not find any other significant differences in speech features between both groups, which may be due to the rather artificial nature of the task not allowing for spontaneous speech to be affected. However, other speech features were relevant for improving prediction accuracy. We were able to predict whether a person would be below or above cut-off with very high accuracy (i.e., 93%). The features that significantly contributed to explained variance in the binary logistic regression model were previously found to be associated with depressive symptoms (e.g., temporal (speech ratio), spectral (MFCC), and prosodic (F0) features; see Table 3) [5, 32–34]. Our results therefore support existing findings [35] and indicate that even in a non-clinical sample, similar speech patterns may be important predictors of depressive symptoms. We similarly achieved good performance (i.e., low mean error) when predicting the actual score in the depression questionnaire. We used random forest regression, which has been used previously to predict depression scores based on speech patterns [36, 37]. These findings may contribute to an early identification of people at risk of developing depression and may allow timely preventive measures. This would, however, require a longitudinal study design in a future study.

Finally, we tested whether we would find significant differences in the Trail Making Test between the above cut-off and the below cut-off group. In addition, we correlated depression scores with the time it took to complete the Trail Making Test. We did not find significant differences between both groups regarding performance but there was a strong positive correlation between time to complete part A of the test and depression scores in the above cut-off group (Fig. 2). Here, individuals with higher depression scores were slower in completing the test. This indicates that those with higher depression scores had deficits in controlling attention or, at least, a reduction in information processing speed. Interestingly, we did not find a correlation between depression scores and time to complete the Trail Making Test B, a measure for attention and executive functions. In manifest depression, both attentional deficits and problems with executive functions are common [38–40]. Comparable to a change in the number of words along the dimensionality of depressive symptoms, also cognitive deficits seem to change along this dimensionality, with attentional deficits appearing very early and problems with executive functions emerging only with manifest depression. This may indicate that attentional deficits are among the first to be associated with symptoms of depression and once depression is clinically manifest, executive functions deficits follow.

Interestingly, in our non-clinical sample, attentional deficits or slower perceptual processing were related to the amount of depressive symptoms, while others reported that attentional deficits in patients with manifest depression were related to illness duration [38]. This may suggest that, once depression becomes manifest, attentional deficits are more related to the accumulating burden of illness than to the severity of the current episode. This, again, points to a qualitative change between depressive symptoms and manifest depression, reflected by diverging speech patterns as well as cognitive deficits.

Our study may have several limitations. First, the speech recordings were relatively short and may have been not long enough for an identification of salient speech features associated with signs of depression. Future studies may consider longer natural speech tasks, particularly in non-clinical samples to test whether the patterns we identify in this study can be replicated. Second, our study sample consisted only of University students which are not representative for the general population. Thus, our results may have been different if we had included participants with different educational backgrounds. Third, we did not do a clinical interview and therefore, we cannot rule out a clinical diagnosis of depression in any of our participants. However, the mean score in those above cut-off was only slightly higher than the cut-off of 22. Therefore, it seems very unlikely that any of our participants suffered from a major depressive episode.

## Conclusions

Taken together, our study adds to the current literature that speech features are sensitive for the detection of depressive symptoms even in a non-clinical sample. In a future longitudinal study, it may be tested whether these are early and sensitive features in individuals at risk of developing depression.

## Supplementary Information

**Additional file 1:Table S1.** Overview of extracted speech features. **Table S2.** Overview of extracted transcript features.

## Data Availability

The datasets used and/or analysed during the current study are available from the corresponding author on reasonable request.
