# Supplementary material for: Detecting subtle signs of depression with automated speech analysis in a non-clinical sample
Source: BMC Psychiatry. 2022 Dec 27;22:830. doi: 10.1186/s12888-022-04475-0 (PMC9793349; doi:10.1186/s12888-022-04475-0)
Supplement: Supplementary file 1 — Additional file 1:Table S1. Overview of extracted speech features. Table S2. Overview of extracted transcript features. [file 12888_2022_4475_MOESM1_ESM.docx]

# Supplementary Material

**Table S1.** Overview of extracted speech features.

| **Speech features** | **Explanation** |
| --- | --- |
| Speech ratio | Prosodic feature for verbal fluency from speech to non-speech proportion in the audio |
| Speech interval | Speech segments uninterrupted by pauses between syllables to measure speech production efficiency |
| Harmonics to noise ratio | Ratio between periodic and non-periodic components of speech which reflects voice quality |
| Sound to noise ratio | Ratio between the power of speech signal and the power of background noise that reflects voice quality |
| F_0_ mean | Mean value of the fundamental frequency in voiced parts of the audio. Fundamental frequency. or pitch. quantifies speech signal’s periodic components for speech production assessment |
| F_0_ standard deviation | Standard deviation of fundamental frequency in voiced parts of the audio |
| Total phonation time | Total time duration of all words across all sentences |
| Number of pauses | Total number of pauses longer than a time threshold between the syllables |
| Apq5 shimmer | Five-point Amplitude Perturbation Quotient, the average absolute difference between the amplitude of a period and the average of the amplitudes of it and its four closest neighbours, divided by the average amplitude |
| Local shimmer | Average absolute difference between the amplitudes of consecutive periods, divided by the average amplitude |
| Average Mel-frequency cepstrals (MFCC) | Decomposition of Mel-frequency cepstral (MFCC) into a range of spectrum coefficients 1 through 20  MFC represents the short-term power spectrum of a sound |
| Deltas | First derivative of the average MFCC values that presents the change in the power spectrum |
| Delta deltas | First derivative of the average MFCC values that presents the rate of change in the power spectrum |
| Espinola zero crossing metric | Measure for the rate at which speech signal crosses the zero reference and its deviation from the reference |
| Average amplitude change | Variation of the signal amplitude over time |
| Amplitude mean absolute value | Variation of the signal amplitude over time without change direction |
| Amplitude third moment | Skewness of the signal amplitude over time |
| Amplitude fourth moment | Kurtosis of the signal amplitude over time |
| Max amplitude | Measure for the maximum disturbance of the air caused by the speech signal |
| Peak frequency | Frequency of the maximum power value in signal frequency spectrum |
| Power spectrum ratio | Power of the most powerful frequency relative to all other frequencies |
| Mean power | Mean power transmitted by the signal |
| Total power | Total power transmitted by the signal |
| Local jitter | Jitter represents the frequency variation between two consecutive periods  Local jitter is the average absolute difference across consecutive cycles normalized by the average period |
| Absolute jitter | Average absolute difference across consecutive cycles in seconds |
| DDP jitter | Average absolute difference between differences across consecutive cycles normalized by the average period |
| PPQ5 jitter | Five-point Period Perturbation Quotient (RPQ) measured by the average absolute difference between a period and the average of it and its four closest neighbours. normalized by the average period |
| RAP jitter | Relative Average Perturbation (RAP). the average absolute difference between a period and the average of it and its two neighbours. normalized by the average period |

**Table S2.** Overview of extracted transcript features.

| **Transcript features** | **Explanation** |
| --- | --- |
| Mean utterance distance | Proxy for linguistic productivity that is the average distance between the utterance in the transcript |
| Noun to verb ratio | Proxy for syntactic complexity measured by the proportion of nouns to verbs in the transcript excluding modals and auxiliaries |
| Adverb rate | Proxy for content richness computed by dividing the number of adverbs to that of other parts-of-speech |
| Verb rate | Proxy for content richness computed by dividing the number of verbs to that of other parts-of-speech |
| Noun rate | Proxy for content richness computed by dividing the number of nouns to that of other parts-of-speech |
| Adjective rate | Proxy for content richness computed by dividing the number of adjectives to that of other parts-of-speech |
| Pronoun rate | Proxy for content richness computed by dividing the number of pronouns to that of other parts-of-speech |
| Conjunction rate | Proxy for content richness computed by dividing the number of conjunctions to that of other parts-of-speech |
| Proper noun rate | Proxy for content richness computed by dividing the number of proper nouns to that of other parts-of-speech |
| Determiner rate | Proxy for content richness computed by dividing the number of determiner to that of other parts-of-speech |
| Adposition rate | Proxy for content richness computed by dividing the number of adpositions to that of other parts-of-speech |
| Average dependency distance | Proxy for syntactic complexity measured by averaging the linear distances between pairs of syntactically related words in the sentence which are represented by different arcs in a dependency tree |
| Total dependency distance | Proxy for syntactic complexity measured by the total distance spanning across the arcs in all dependency trees |
| Average dependencies | Proxy for syntactic complexity measured by the average number of dependency arcs in all dependency trees |
| Average number of subordinate clauses | Proxy for sentence complexity measured by the average number of subordinate clauses per sentence |
| Number of semantic clusters | Proxy for semantic consistency measured by the number of groups formed by semantically similar words |
| Mean cluster size | Proxy for semantic consistency computed by averaging the largest distances from the clusters of semantically similar words |
| Standard deviation of cluster size | Variation in sizes of clusters made up of semantically similar words |
| Tangentiality score | Proxy for content disconnectedness and topic inconsistency of a discourse |
| Coherence score | Proxy for discourse coherence measured by the similarity between consecutive sentences |
| Mean cluster density | Proxy for semantic consistency measured by averaging the number of words in all semantic clusters |
| Largest cluster density | Number of words that make up the largest semantic cluster |
| Number of cluster switches | The number of transitions patient makes between semantic clusters that captures cognitive flexibility |
